# The Use of Metal/ZSM-5 Nanosheet for Efficient Catalytic Cracking of Cross-Linked Polyethylene for High-Voltage Cable Insulation

**DOI:** 10.3390/ma18204675

**Published:** 2025-10-11

**Authors:** Zhenfei Fu, Yuqi Pan, Rui Wang, Shilong Suo, Zheng Wang, Xiangyang Peng, Pengfei Fang

**Affiliations:** 1 Key Laboratory of Nuclear Solid State Physics Hubei Province, School of Physics and Technology, Wuhan University, Wuhan 430072, China; 2 Guangdong Key Laboratory of Electric Power Equipment Reliability, Electric Power Research Institute of Guangdong Power Grid Co., Ltd., Guangzhou 510080, China

**Keywords:** XLPE cracking, ZSM-5 nanosheets, metal ion loading, acidity regulation, low-carbon olefins, catalytic cracking

## Abstract

Cross-linked polyethylene (XLPE) has been widely used in high-voltage cables due to its superior properties, but its thermoset cross-linked structure makes it difficult to recycle. Catalytic pyrolysis offers a feasible pathway for converting XLPE into high-value chemicals. In this study, a systematic study on the catalytic cracking of XLPE using metal ion-loaded ZSM-5 nanosheets was conducted, and ZSM-5 nanosheets loaded with Ag, Mo, Ni, and Ce were prepared via ion exchange. After metal loading, ZSM-5 retained the MFI framework structure, but the specific surface area and mesopore volume varied depending on the type of metal. Temperature-Programmed Desorption of Ammonia results indicated that metal–support interactions enhanced the acidity of ZSM-5. Among the catalysts, Ag-loaded ZSM-5 exhibited the highest efficiency: with 10 wt% Ag, at 380 °C, the conversion reached 94.1%, with 52.5% light olefins in the gas phase and 59.4% benzene, toluene, and xylene (BTX) in the liquid products. Further studies on different Ag loadings revealed that moderate Ag loading (5 wt%) provided the best overall balance, maintaining 92.3% conversion, 56.1% selectivity to light olefins, and 58.2% BTX in the liquid fraction. These findings demonstrate that tuning the metal loading effectively optimizes the acidity and pore structure of ZSM-5, thereby enabling controlled regulation of XLPE pyrolysis product distribution.

## 1. Introduction

High-voltage cables are an essential component of power systems, and their usage continues to rise with global electricity demand. However, such cables have a finite design lifespan, and as increasing numbers reach end-of-life, large-scale recycling becomes a pressing issue. Cross-linked polyethylene (XLPE), the principal insulating material in high-voltage cables, poses significant recycling challenges due to its thermosetting cross-linked structure because of its thermosetting cross-linked structure [1]. Direct landfilling or incineration causes severe waste and pollution, which contradicts current low-carbon and energy-saving goals. To address this, various recycling methods have been developed, including thermomechanical shearing and plasticization [2], ultrasound-assisted extrusion [3], solid-state shear pulverization [4], supercritical decrosslinking [5], and chemical recycling [6,7,8]. Among these, catalytic cracking—a form of chemical recycling—stands out as it efficiently converts polyolefins into high-value-added products such as light olefins (ethylene, propylene, butene) and light aromatics (benzene, toluene, xylene). Compared with thermal cracking, catalytic cracking usually proceeds under milder conditions with higher selectivity [9].

For polyethylene (PE) materials, acidic catalysts are typically employed to crack them into high-value light olefins and aromatics [10]. Previous studies have shown that during catalytic cracking of polyethylene, long-chain polymers adsorb onto Brønsted acid sites of the catalyst, undergo protonation to form carbocation intermediates, which subsequently crack into smaller carbocations [11]. These carbocations subsequently rearrange, forming isomerized carbocations. Next, carbocations undergo four types of β-scission, generating olefins, alkanes, and aromatic hydrocarbons [12]. However, the efficiency and selectivity of polyolefin cracking are strongly affected by catalyst structure and acidity, as well as adsorption, desorption, and diffusion of feedstocks, intermediates, and products on acidic sites [13].

ZSM-5 zeolite, due to its unique Mobil Five (MFI) channel system, tunable acid sites, and excellent thermal stability, exhibits distinct advantages in polyolefin cracking [14]. For example, Dai et al. systematically studied how acid density and pore structure influence high-density polyethylene (HDPE) cracking over prepared or modified ZSM-5 zeolites [15]. Tarach et al. revealed that hierarchical ZSM-5 shows higher activity and resistance to deactivation in low-density polyethylene (LDPE) cracking [16]. Their further study demonstrated the interplay of pore structure and acidity on product distribution and catalyst deactivation in polypropylene (PP) cracking [17]. The diffusivity and acidity of ZSM-5 play crucial roles in polyolefin catalytic cracking. Diffusion is mainly governed by morphology, while acidity can be regulated by several factors, including Si/Al ratio and incorporation of extra-framework elements. The MFI topology consists of straight channels along the [010] direction and sinusoidal channels along [100]; diffusion in straight channels is faster than in sinusoidal ones [18]. Therefore, developing short b-axis ZSM-5 nanosheets is an effective strategy to enhance cracking performance [19]. For instance, Dou et al. reported that nanosheet ZSM-5 provided high propylene selectivity and long lifetimes in methanol to propylene (MTP) [20]. Ryoo et al. demonstrated that ultrathin ZSM-5 nanosheets (2 nm) deactivated more slowly in methanol to gasoline (MTG) compared with conventional ZSM-5 [21]. Zhang et al. found that ZSM-5 nanosheets achieved 90.5% conversion in cracking 1,3,5-triisopropylbenzene, 5.76 times higher than conventional ZSM-5 [22]. Adjusting the Si/Al ratio directly influences acidity and acid site distribution: lower Si/Al ratios increase total acidity and Brønsted site density, but excessively low ratios risk framework collapse [23,24]. In addition to Si/Al tuning, incorporating framework or extra-framework metal species is another key strategy to modulate acidity [25,26]. Ion exchange, based on electrostatic interactions, is a simple and efficient method to introduce extra-framework metals [27]. Ce, Mo, Ni, and Ag are frequently used to enhance selectivity toward light products [28,29,30]. For example, Wang et al. reported Ce-loaded ZSM-5 nanosheets achieving 96.3% conversion, 80.9% C_3_–C_5_ selectivity, and excellent stability in LDPE hydrocracking [31]. Sun et al. showed Mo-loaded ZSM-5 delivered 67.4% selectivity in biomass cracking [32]. Zhang et al. synthesized Ni-loaded ZSM-5 catalysts, with the best 10 wt% Ni sample yielding up to 59.2% benzene, toluene, and xylene (BTX) from PE cracking [33]. Zhang et al. also reported Ag-loaded ZSM-5 achieving a 67.55 wt% conversion of n-pentane [34].

However, research on metal-loaded ZSM-5 in XLPE cracking remains scarce. Based on this, the present study employed short b-axis ZSM-5 nanosheets with an appropriate Si/Al ratio (40) as the host, introducing Ni, Ce, Mo, and Ag via ion exchange. Using X-Ray diffraction (XRD), scanning electron microscopy (SEM), transmission electron microscopy (TEM), Brunauer–Emmett–Teller (BET), X-Ray photoelectron spectroscopy (XPS), and ammonia temperature-programmed desorption (NH_3_-TPD), we systematically analyzed structural characteristics and acid distribution. Furthermore, catalytic cracking experiments were conducted to evaluate product distributions, and the effects of different Ag loading amounts on catalytic performance were investigated.

## 2. Results and Discussion

### 2.1. ZSM-5 Loaded with Different Metal Ions (Ce, Mo, Ni, and Ag)

The detailed synthesis procedure of ZSM-5 is provided in the Supplementary Materials, and the synthesis scheme is shown in Figure S1.

#### 2.1.1. Catalyst Characterization

**Figure 1:**
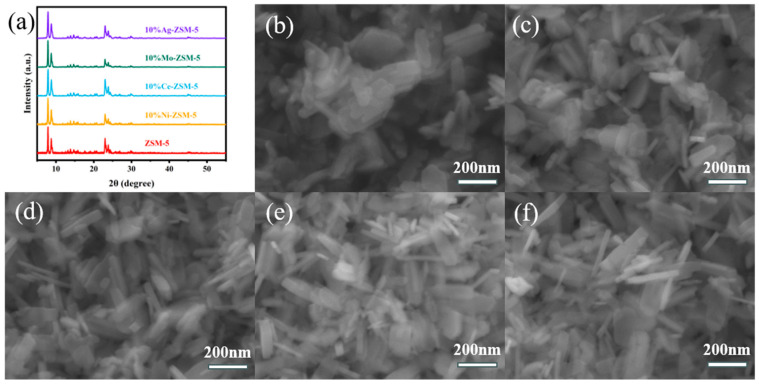
(**a**) XRD spectra of ZSM-5 loaded with different metal ions and their SEM images: (**b**) ZSM-5, (**c**) 10%-Ni-ZSM-5, (**d**) 10%-Ce-ZSM-5, (**e**) 10%-Mo-ZSM-5, (**f**) 10%-Ag-ZSM-5.

To determine whether metal loading affects the crystal structure, XRD and SEM characterizations were conducted on ZSM-5 nanosheets loaded with different metals. Figure 1a shows the XRD patterns of the samples. ZSM-5 nanosheets exhibited the typical MFI-type structure, with distinct diffraction peaks at 2θ = 7.8°, 8.8°, 23°, 23.8°, and 24.3° [19]. The metal-loaded ZSM-5 nanosheet catalyst exhibits the same characteristic diffraction peaks as the ZSM-5 nanosheets in the XRD pattern, indicating that the metal loading does not alter its topological structure, and the framework characteristics of ZSM-5 nanosheets are retained. Scanning electron microscopy (SEM) was used to determine whether the metal ion loading would affect the morphology of ZSM-5. Figure 1b–f show the SEM images of ZSM-5, 10% Ni-ZSM-5, 10% Ce-ZSM-5, 10% Mo-ZSM-5, and 10% Ag-ZSM-5, respectively. From these images, it can be seen that the morphology of the ZSM-5 nanosheets remained almost unchanged after metal ion loading. They still exhibited the typical elongated hexagonal nanosheet morphology, indicating that the metal ion loading did not disrupt the morphology of the sample, which is consistent with the XRD results.

**Figure 2:**
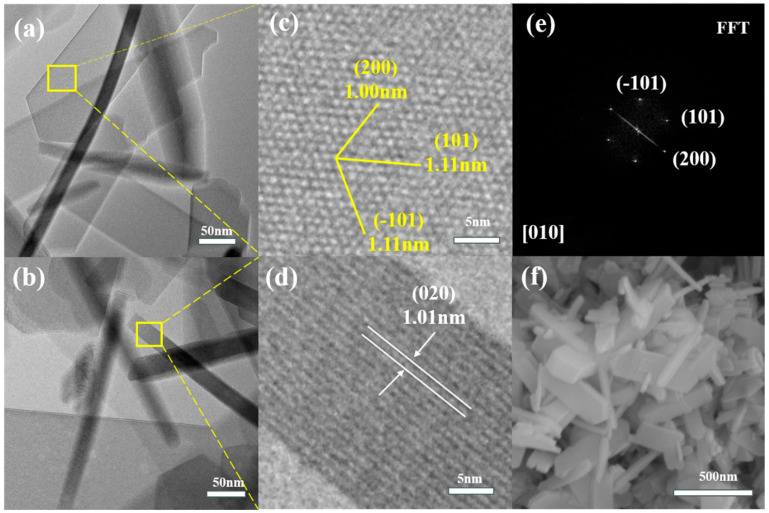
(**a**) TEM, (**c**) HRTEM, and (**e**) FFT images of ZSM-5 nanosheets along the [010] direction; (**b**) TEM and (**d**) HRTEM images along the [100] direction; and (**f**) SEM image.

The HRTEM and FFT images of ZSM-5 are shown in Figure 2. The HRTEM and FFT results indicate that the ZSM-5 nanosheets synthesized in this work are short b-axis nanosheets, with the thinnest thickness along the straight channels.

**Figure 3:**
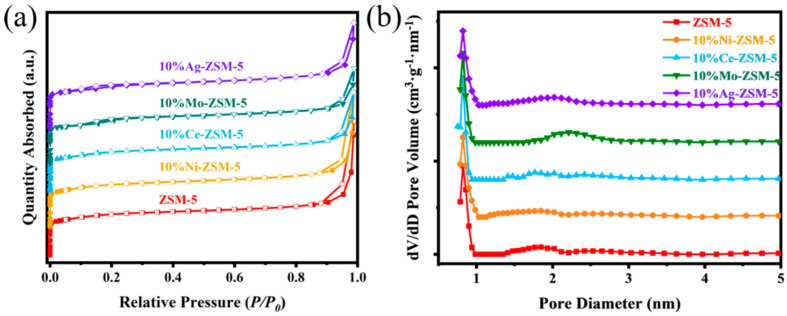
Surface area and pore-size analysis of ZSM-5 nanosheets loaded with different metals: (**a**) N_2_ adsorption–desorption isotherms; (**b**) pore-size distribution.

Figure 3 shows the specific surface area and pore size distribution results for ZSM-5 nanosheets loaded with different metal ions (Ni, Ce, Mo and Ag). Figure 3a displays the nitrogen adsorption–desorption isotherms, and Figure 3b shows the pore size distribution. According to the nitrogen adsorption–desorption isotherms, all samples exhibit typical type I isotherms, and at lower relative pressures (P/P_0_ < 0.1), the nitrogen adsorption significantly increases, indicating that these samples have a typical microporous structure. As the relative pressure increases (0.8 < P/P_0_), nitrogen molecules desorb from the pores. However, due to the pore structure, the nitrogen molecules within the pores cannot immediately desorb and require some time before complete desorption, forming an H3-type hysteresis loop. This indicates the presence of mesoporous structures in the samples. However, compared to the metal-free ZSM-5 nanosheets, the hysteresis loop of the metal-loaded ZSM-5 nanosheets is significantly reduced, indicating that the metal ion loading decreases the number of mesopores in the ZSM-5 nanosheets. Specifically, the loading of Ce and Mo significantly affects the average pore size, reducing it to 0.798 nm and 0.803 nm, respectively. This change suggests that the doping of metals influences the pore structure of ZSM-5 nanosheets, particularly the mesoporous structure. This may be due to the agglomeration of metal ions, causing the blockage of some pores and reducing the openness of the pore channels. The pore size distribution of the metal-loaded ZSM-5 nanosheets was calculated using the DFT method, and the curves further confirm the impact of metal loading on the pore structure of ZSM-5 nanosheets. Specifically, the Ni-loaded sample shows a higher mesopore volume, significantly higher than other metal-loaded samples. The calculated specific surface area and pore size distribution data for ZSM-5 nanosheets with metal loading further support this observation.

From the specific data in Table 1, it can be seen that after the metal loading of Ce, Mo, and Ag, the specific surface area of ZSM-5 nanosheets decreased to varying extents, with values reduced to 392.462 m^2^/g, 390.593 m^2^/g, and 392.621 m^2^/g, respectively. This phenomenon can be attributed to the high charge density of Ce and Mo metal ions, which, during their incorporation into the ZSM-5 framework or pores, tend to induce local charge imbalances, leading to slight structural collapse and a reduction in effective specific surface area and pore volume. Additionally, the agglomeration of metal ions may cause the blockage of some pores, reducing the pore openness. In contrast, Ni loading increased the specific surface area of the nanosheets, indicating that Ni was more effectively dispersed onto the surface and within the pores of the molecular sieve during the ion-exchange process. This behavior is likely related to the suitable ionic radius of Ni^2+^ and its higher exchange efficiency, which enhances the external surface area and mesopore contribution of ZSM-5 nanosheets while maintaining the original crystal structure and optimizing the overall pore structure. As for the Ag-loaded samples, the decrease in specific surface area was relatively moderate. These Ag^+^ ions tend to migrate and sinter on the surface of ZSM-5 nanosheets, forming a coating layer that partially covers the pores, affecting the nitrogen adsorption capacity and reducing both the specific surface area and pore utilization.

**Figure 4:**
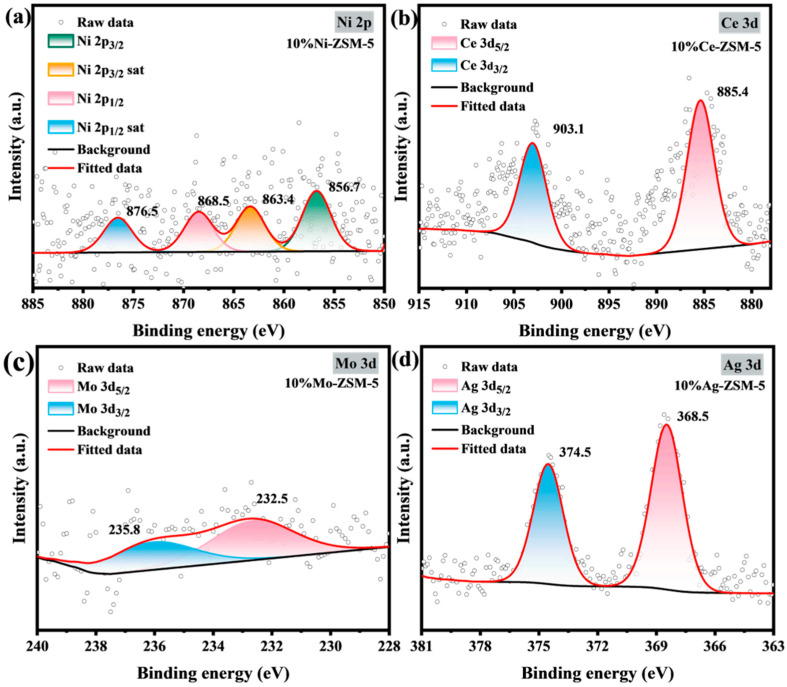
XPS spectra of metal-loaded ZSM-5 nanosheets: (**a**) Ni, (**b**) Ce, (**c**) Mo, (**d**) Ag.

XPS testing and analysis were performed on ZSM-5 nanosheets modified with different metal ions to determine whether the metals were incorporated into the ZSM-5 nanosheets and to examine the distribution of the metal oxidation states within the ZSM-5 nanosheets. The detailed analysis is shown in Figure 4. Figure 4a shows the high-resolution XPS spectrum of Ni-loaded ZSM-5 nanosheets. In the high-resolution XPS spectrum, two distinct characteristic peaks are observed. Peak deconvolution of these two peaks reveals the oxidation state and orbital energy level distribution of the metal Ni. The results show that the Ni 2p orbital binding energy in 10% Ni-ZSM-5 nanosheets exhibits typical characteristics of a high oxidation state. The main peak of Ni 2p_3/2_ appears at 856.7 eV, accompanied by a distinct satellite peak at 863.4 eV. The main peak of Ni 2p_1/2_ is located at 868.5 eV, with the satellite peak at 876.5 eV. These peak positions are consistent with those reported in the literature for nickel ions. Based on this, it can be concluded that the Ni in the sample is predominantly in the +2 oxidation state, indicating that the Ni forms a stable interaction with the ZSM-5 nanosheet support. Similarly, Figure 4b shows the high-resolution XPS spectrum of Ce-loaded ZSM-5 nanosheets. The spectrum shows distinct Ce 3d_5/2_ and Ce 3d_3/2_ characteristic peaks at 885.4 eV and 903.1 eV, respectively, indicating that the loaded Ce is predominantly in the +3 oxidation state. Figure 4c shows the XPS spectrum of Mo-loaded ZSM-5 nanosheets. The spectrum reveals prominent characteristic peaks at 232.5 eV and 235.8 eV, indicating that Mo is primarily in the +6 oxidation state. Figure 4d shows the XPS spectrum of Ag-loaded ZSM-5 nanosheets. The characteristic peaks appear at 368.5 eV and 374.5 eV, corresponding to the Ag 3d_5/2_ and Ag 3d_3/2_ orbitals. These two characteristic peaks are consistent with those reported for AgNO_3_ and Ag-ZSM-5 [35], indicating that the 10% Ag-ZSM-5 nanosheets are predominantly in the +1 oxidation state. Based on the above analysis, it can be concluded that metal ions can be loaded onto ZSM-5 nanosheets using the ion-exchange method.

**Figure 5:**
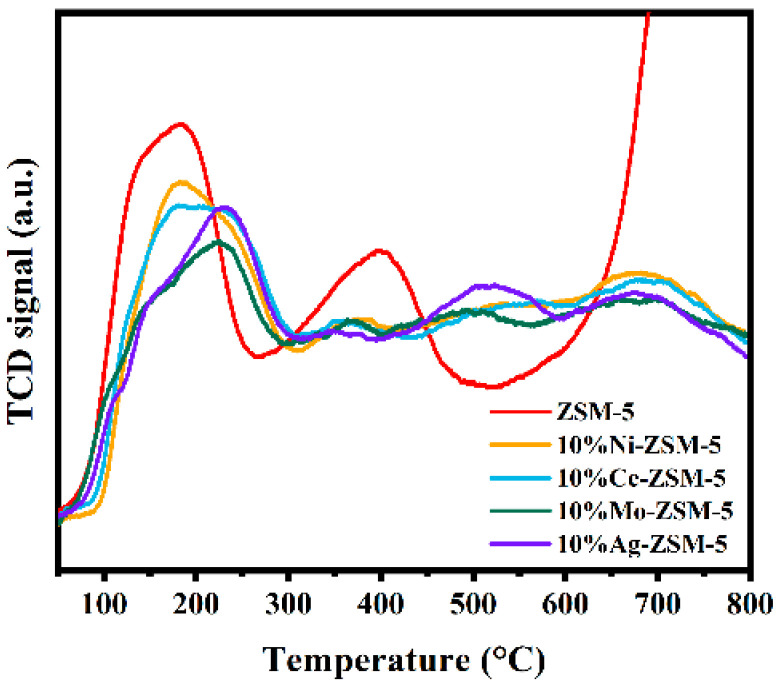
NH_3_-TPD profiles of ZSM-5 nanosheets with different metal loadings.

NH_3_-TPD was used to evaluate acidity (Figure 5). All metal-loaded samples exhibited desorption peaks at 100–280 °C, 300–500 °C, and 500–800 °C, corresponding to weak-to-medium acid sites, strong acid sites, and cooperative sites formed by the interaction between metal and strong acid sites, respectively. Compared with pristine ZSM-5, metal-loaded catalysts showed enhanced high-temperature desorption peaks, indicating improved thermal stability due to metal–support interactions. Ni loading increased weak and medium–strong acidity, consistent with electronic interactions between Ni d-orbitals and Si–O–Al bonds. Ce loading, owing to its redox properties, improved acid site distribution and suppressed overcracking. Mo loading introduced new weak acid sites by forming Mo–O–Al linkages. Ag loading reduced high-temperature desorption intensity, as Ag^+^ partially replaced protons at Brønsted sites, forming new acid centers. Table 2 lists the amounts of different types of acid sites in the various catalysts. The data indicate that metal loading exhibits significant differences in terms of acid site types and acid strength distribution. The changes in acidity are mainly attributed to the coverage of acid sites during the metal loading process and the formation of metal–support interactions. It can be observed that the loading of metals Ni and Ce increases the weak–medium acid sites in ZSM-5 nanosheets, providing more electron-accepting sites.

#### 2.1.2. Catalytic Cracking Performance

The specific reaction conditions are provided in the Supplementary Materials, the schematic of the reaction setup is shown in Figure S2, the chromatogram of the main gaseous product peaks is shown in Figure S3, and the chromatogram of the main liquid product peaks is shown in Figure S4.

**Figure 6:**
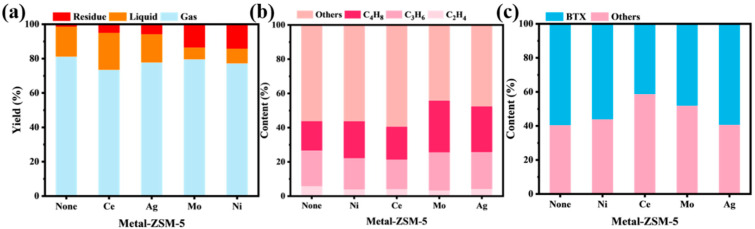
Catalytic cracking of XLPE over different metal ion-loaded ZSM-5 nanosheets: (**a**) product distribution, (**b**) gas selectivity, and (**c**) liquid selectivity.

Figure 6a shows the product distribution of catalytic cracking of XLPE using ZSM-5 nanosheets before and after loading different metal ions (Ce, Ni, Mo, Ag). As can be seen from the figure, compared to the metal-free ZSM-5 nanosheets, the metal-loaded modified samples show a certain degree of decrease in gas yield. Among them, the catalyst without metal loading has the highest gas yield, reaching 81.1%, and the residual yield is the lowest, at only 1.5%, indicating its optimal catalytic cracking activity. The catalysts loaded with Ni and Mo showed a significant decrease in conversion rate, with the residual yield increasing to 14.3% and 13.7%, and the gas yield dropping to 77.2% and 79.5%, respectively. In contrast, the catalyst loaded with Ce maintained a relatively high conversion rate (residual yield of 5.07%), while the liquid yield increased to 21.5%, demonstrating its potential for product distribution control. The redox properties of Ce and its modulation of acidic sites likely inhibited deep cracking reactions, allowing more intermediate products to be retained and concentrated as liquid products. Ce primarily adjusts the strength of the adjacent strong acid sites through electronic effects, increasing the number of weak and medium-strength acid sites, inhibiting excessive cracking, and enhancing the yield of liquid oil. On the other hand, the sample loaded with Ag ions maintains a high conversion rate (94.1%) while exhibiting a higher gas yield (77.7%), as the Ag ion-loaded sample has moderate acidity.

Figure 6b shows the distribution of gaseous product components from the catalytic cracking of XLPE using ZSM-5 nanosheets modified with different metal ions. As can be seen from the figure, the catalysts loaded with Mo and Ag exhibit a significantly higher total proportion of light olefins in the cracking products compared to the metal-free and other metal-modified samples. The content of C_4_H_8_ reaches its highest value of 30.3% in the Mo-loaded sample. This increase can be attributed to the role of metal loading in modulating the acidity distribution of ZSM-5 nanosheets. Specifically, after loading Mo and Ag, the weak to medium-strong acid and strong acid sites in the catalysts were reduced compared to other metal-loaded samples. This effectively avoided the intensification of deep cracking and hydrogen transfer reactions while promoting dehydrogenation reactions, thereby enhancing the selectivity of light olefins. In contrast, the Ce-modified catalyst showed a decreasing trend in the proportion of light olefins in the gaseous products, with the ethylene and propylene contents dropping to 4% and 17.2%, respectively. This phenomenon is mainly due to the significant increase in medium-strong acid sites with Ce loading, which promoted hydrogen transfer and cyclization reactions, leading to the formation of high molecular weight intermediate products such as aromatics and saturated alkanes, while suppressing the formation of light olefins.

Figure 6c shows the distribution of BTX in the liquid products obtained from the catalytic cracking of XLPE using ZSM-5 nanosheets modified with different metal ions. It can be observed that the metal-free ZSM-5 nanosheet catalyst exhibited the highest BTX yield, reaching 59.6%, indicating its inherent excellent aromatization ability. The BTX proportion in the liquid products from Ag-loaded ZSM-5 nanosheets remained almost unchanged at 59.4%, which can be attributed to the fact that Ag loading did not significantly alter the pore structure of the molecular sieve, and its relatively mild acidity regulation preserved the good selectivity of ZSM-5 towards aromatics. In contrast, ZSM-5 nanosheets loaded with Ce and Mo exhibited a significant decrease in BTX selectivity, with values of 42.1% and 48.2%, respectively. This can be primarily attributed to the introduction of Ce and Mo, which hindered the aromatization reaction to some extent: on one hand, these two metals have larger ionic radii and stronger metal–support interactions, causing some degree of structural disturbance in the molecular sieve pores, thus impeding the diffusion of aromatic molecules; on the other hand, Ce loading significantly increased the number of medium-strong acid sites, promoting deeper cracking reactions but possibly inhibiting the stable formation and accumulation of aromatic intermediates. Additionally, Mo loading might introduce some neutral or weak acid sites, which could reduce the efficiency of the aromatization reaction. Notably, although the Ni-loaded sample showed a slightly lower total conversion rate, its BTX proportion reached 56.1%, exhibiting relatively good aromatization performance. This may be attributed to the excellent dehydrogenation performance of Ni, which facilitated the conversion of chain-like intermediates into aromatic compounds while maintaining the support role of ZSM-5’s framework acidity in the aromatization reaction.

### 2.2. Ag-Ion Loading at Different Ratios on ZSM-5 Nanosheets

Through a systematic analysis of the catalytic cracking performance of ZSM-5 nanosheet catalysts modified by loading different metals (Ni, Ce, Mo, Ag), it was found that Ag-loaded ZSM-5 nanosheets exhibited well-balanced and superior catalytic characteristics in multiple aspects, making them the preferred choice for further in-depth investigation. In terms of overall catalytic cracking efficiency, the Ag-loaded ZSM-5 nanosheets showed a relatively high conversion rate with the least amount of residue formation, indicating that their catalytic activity remained strong without being significantly weakened by the introduction of metal species. Regarding product distribution, this catalyst achieved both a high proportion of gaseous products and a satisfactory yield of liquid oil, demonstrating excellent product regulation capability. Moreover, in the composition of gaseous products, the Ag-loaded sample maintained a high proportion of light olefins, while in the liquid products, it exhibited sound BTX selectivity. This suggests that the catalyst not only effectively promotes cracking but also possesses strong ability to suppress side reactions.

#### 2.2.1. Catalyst Characterization

Figure S5 presents the XRD patterns and SEM of ZSM-5 nanosheets with different Ag loadings. All samples retained the characteristic diffraction peaks of the MFI framework, indicating that the overall zeolite structure was preserved after Ag incorporation. No distinct Ag-related peaks were detected, suggesting high dispersion of Ag within the nanosheets. Figure S5b–f shows SEM images of ZSM-5, 1%Ag-ZSM-5, 5%Ag-ZSM-5, 10%Ag-ZSM-5, and 15%Ag-ZSM-5. The morphology remained essentially unchanged after metal loading, with all samples exhibiting the characteristic elongated hexagonal nanosheet shape, confirming that metal loading did not disrupt morphology.

**Figure 7:**
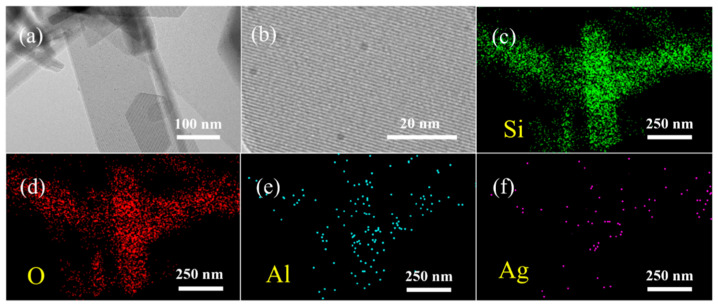
5%Ag-ZSM-5 nanosheets: (**a**,**b**) TEM images; (**c**–**f**) EDS elemental maps of Si, O, Al, and Ag.

Figure 7a,b show the TEM images of the 5% Ag-ZSM-5 nanosheet sample. Figure 7a clearly demonstrates that the sample retains a regular hexagonal prismatic structure, indicating that the metal loading did not disrupt the original morphological structure of the ZSM-5 nanosheets. Further observation of Figure 7b reveals noticeable defect structures on the surface of the nanosheets, which may be attributed to the Ag loading. This could be due to the replacement of protons at the original Brønsted acid sites by Ag^+^ ions. Figure 7c–f show the EDS element distribution maps for the 5% Ag-ZSM-5 nanosheet sample, displaying the spatial distribution of Si, O, Al, and Ag elements. As can be seen from the images, the Si, O, and Al elements are uniformly distributed within the nanosheets, reflecting the structural integrity of the MFI framework. Meanwhile, the Ag element is highly dispersed across the entire surface of the catalyst without obvious agglomeration, further confirming the excellent distribution of Ag on the ZSM-5 nanosheet support.

**Figure 8:**
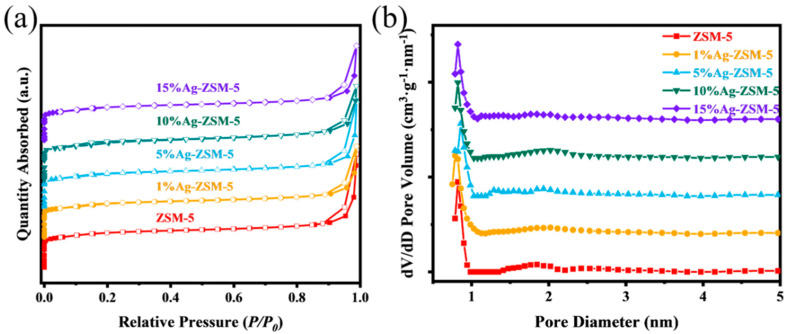
Surface area and pore-size analysis of ZSM-5 nanosheets with different Ag loadings: (**a**) N_2_ adsorption–desorption isotherms; (**b**) pore-size distribution.

Figure 8 shows the nitrogen adsorption–desorption isotherms and pore size distribution curves for ZSM-5 nanosheets with different Ag loadings. First, from the nitrogen adsorption–desorption isotherms, it can be observed that all samples exhibit typical type I isotherm characteristics, with the adsorption rapidly increasing in the low-pressure region, indicating that they primarily have a microporous structure. At the high relative pressure region (P/P_0_ > 0.8), a distinct H3-type hysteresis loop is observed, trending towards type IV isotherm, which indicates the presence of a certain amount of mesopores in the samples. Therefore, the different Ag loadings did not disrupt the micropore-mesopore composite structure of the ZSM-5 nanosheets. Further analysis of the pore size distribution curves reveals that the 5% Ag sample has a strong distribution peak around 0.5–1.0 nm. For zeolite catalysts, both the BET surface area and the pore size decrease after metal ion loading, which may be due to the accumulation of metal atoms [31], Moreover, with increasing metal loading, the reduction in average pore size may result from the partial blockage of the microporous structure by the metal ions [36,37].

From the data in Table 3, it can be seen that the specific surface area decreases as the Ag loading increases. The specific surface area of the metal-free sample is 419.440 m^2^/g, which slightly decreases to 398.798 m^2^/g after loading 1% Ag. This decrease is likely due to the local accumulation of silver on the surface or in the pores of the molecular sieve, which hinders gas molecules from entering some of the pores. When the loading increases further, the specific surface area shows no significant change, indicating that excess Ag might distribute on the outer surface with a larger particle size. Additionally, from the changes in mesopore volume and total pore volume, it can be seen that an appropriate Ag loading effectively eliminates the pore size changes caused by low Ag loadings.

**Figure 9:**
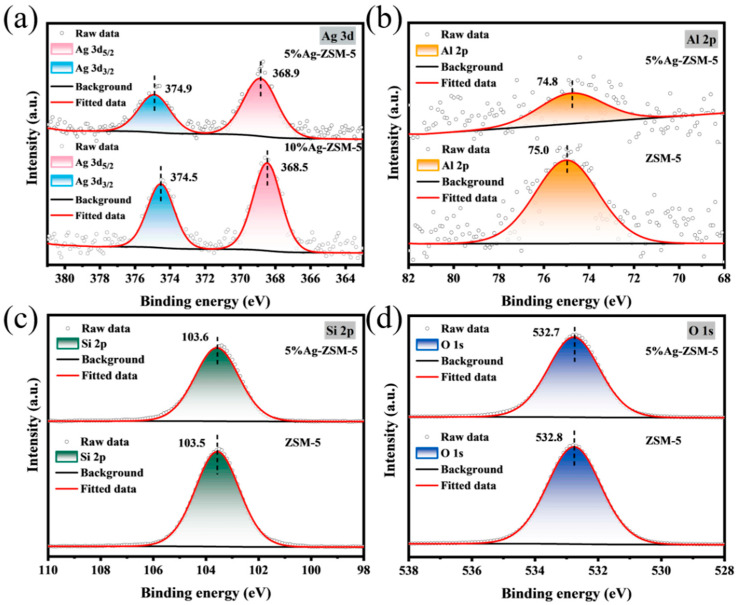
(**a**) XPS spectra of 5% and 10%Ag-ZSM-5 nanosheets; comparison of (**b**) Al 2p, (**c**) Si 2p, and (**d**) O 1s for 5%Ag-ZSM-5 vs. pristine ZSM-5.

High-resolution XPS spectra of 5%Ag-ZSM-5 and 10%Ag-ZSM-5 (Figure 9a) showed typical Ag 3d_5/2_ and Ag 3d_3/2_ peaks at 368–375 eV, confirming silver in a predominantly Ag^+^ state. For 5%Ag-ZSM-5, peaks were at 368.9 eV and 374.9 eV; for 10%Ag-ZSM-5, they shifted slightly to 368.5 eV and 374.5 eV. The spin–orbit splitting was 6.0 eV, consistent with Ag^+^. The slight shift is attributed to Ag dispersion and particle size effects. At 5% loading, Ag was more highly dispersed, causing electron redistribution and slightly higher binding energy, while at 10% loading, partial aggregation led to lower binding energy.

Figure 9b–d compare Al 2p, Si 2p, and O 1s spectra for 5%Ag-ZSM-5 and pristine ZSM-5. Both exhibited clear peaks, confirming framework integrity. Although the differences are small, all orbital binding energies show varying degrees of redshift, indicating that the silver loading has altered the electronic distribution of the Si-O-Al structure in the framework to some extent. The slight shifts in these energy levels can be attributed to the electronic coupling between Ag and the oxygen or silicon atoms in the ZSM-5 framework. In particular, Ag in a highly dispersed state is more likely to exchange electrons with the framework surface, leading to chemical shifts. This electronic interaction may not only introduce new Lewis acid sites around the framework but also alter the acidity of the catalytic active sites by modulating the electron density, thereby affecting catalytic activity. Meanwhile, we listed the atomic ratios obtained from XPS in Table S2. For the ion-exchange method, metal ions are generally loaded at shallow depths, so the Ag atomic ratio given by XPS only reflects the surface atomic ratio, which is much higher than the overall proportion. Both the EDS mapping and the XPS atomic ratios confirm that Ag atoms are distributed relatively uniformly on the surface of the ZSM-5 nanosheets.

**Figure 10:**
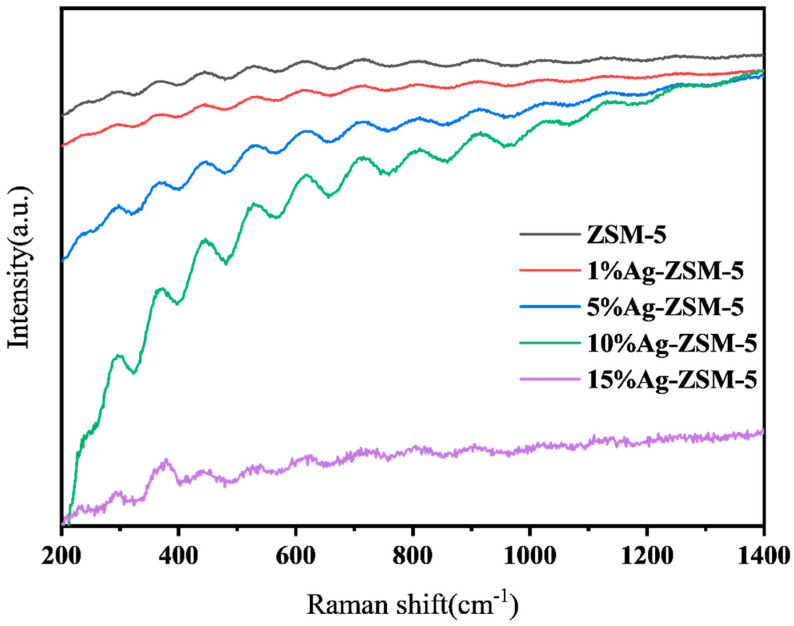
Raman spectra of ZSM-5 nanosheets loaded with Ag ions at different ratios.

Figure 10 presents the Raman spectra of ZSM-5 nanosheets and Ag-loaded ZSM-5 with different loadings. Since the Raman peak of the Ag-O coordination bond is relatively weak, no distinct additional peaks are observed in the spectra of 1%, 5% and 10% Ag-ZSM-5 samples. In contrast, the Raman peak appearing at 385 cm^−1^ in the 15%Ag-ZSM-5 sample can be attributed to the formation of AgO, resulting from the surface oxidation of excessive Ag metallic particles [38].

**Figure 11:**
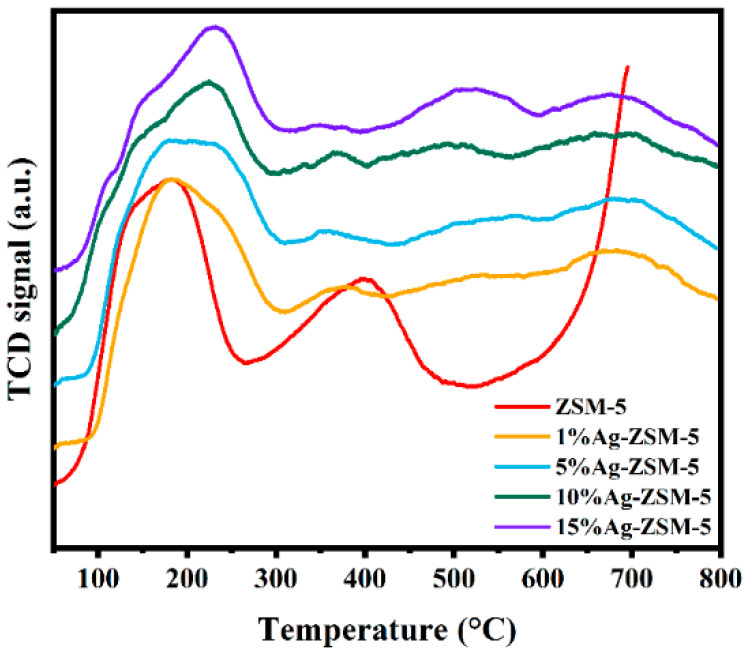
NH_3_-TPD profiles of ZSM-5 nanosheets with different Ag loadings.

Figure 11 shows the NH_3_-TPD curves of ZSM-5 nanosheets with different Ag loadings. From the figure, it can be seen that all loaded samples exhibit three main NH_3_ desorption peaks in the range of 100–800 °C, corresponding to weak to medium-strong acid, strong acid, and metal-strong acid synergistic acid sites, indicating the presence of abundant acidic centers on their surfaces. Compared to the Ag-free ZSM-5 sample, the desorption peaks of the Ag-modified samples shift overall to higher temperatures, suggesting that the introduction of Ag enhances the thermal stability of the acid sites. However, as shown in Table 4, the intensity of the peak corresponding to strong acid sites significantly decreases after Ag loading, especially for the 10% and 15% loaded samples, showing a noticeable decline. This trend suggests that Ag doping partially occupies the proton sites of the Si-O(H)-Al structure in the ZSM-5 framework, forming Al-O-Ag bonds and weakening the original Brønsted acid centers. As the Ag loading increases, this substitution effect becomes more significant, leading to a simultaneous decrease in the total acidity and the number of strong acid sites. Furthermore, as the Ag loading increases, the proportion of strong acid sites in ZSM-5 nanosheets decreases, which is due to the covering effect of Ag on the strong acid centers. This may be related to the agglomeration of Ag particles on the molecular sieve surface, causing uneven distribution of acid sites or partial shielding. In summary, Ag loading not only enhances the thermal stability of the acid sites in ZSM-5 nanosheets but also influences the selectivity and reaction activity in the catalytic cracking process by altering the distribution and strength of the acid sites.

#### 2.2.2. Catalytic Cracking Performance

In the previous section, the catalytic cracking performance of ZSM-5 nanosheets loaded with four different metals was compared, among which Ag-modified ZSM-5 demonstrated favorable catalytic activity and high selectivity toward light olefins. Based on this, the effect of different Ag loadings on the catalytic performance of ZSM-5 nanosheets was further investigated, as shown below.

**Figure 12:**
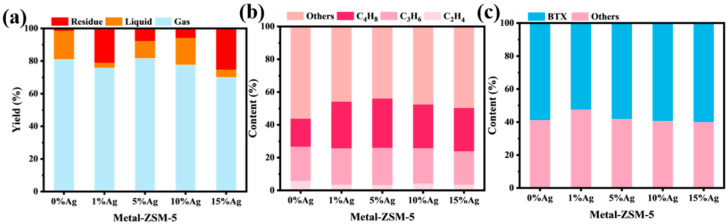
Catalytic cracking of XLPE over ZSM-5 nanosheets with different Ag loadings: (**a**) product distribution, (**b**) gas selectivity, and (**c**) liquid selectivity.

Figure 12a show the product composition distribution of XLPE catalytic cracking using ZSM-5 nanosheets with different Ag loadings. As shown in the figure, the Ag-free ZSM-5 nanosheets exhibit the best cracking activity, with an XLPE conversion rate as high as 98.5%, where the gas and liquid product yields are 81.1% and 17.4%, respectively, and only 1.47% of residual products are generated. As the Ag loading increases, the overall catalytic activity of the catalyst shows a trend of increasing first and then decreasing. At a 1% Ag loading, the gas product yield decreases to 75.9%, and the residual product significantly increases, with the conversion rate dropping to about 79%. When the loading increases to 5%, the catalytic performance improves significantly, with the gas product yield rising to 81.7% and the residual product proportion decreasing to 7.7%, indicating that the catalyst at this loading has good cracking activity and product selectivity. Further increasing the Ag loading to 10% and 15% leads to a decline in catalytic performance, with the residual product proportion significantly rising, and both gas and liquid product yields decreasing. Particularly at 15% loading, the residual product reaches 25.3%, and the conversion rate drops to about 75.5%. This change trend is primarily attributed to the regulation of the acidity structure of ZSM-5 nanosheets by Ag. The introduction of an appropriate amount of Ag facilitates electron transfer between metal–support interactions and the framework acidic sites, weakening the acidity of the strong acid sites, which helps to regulate the cracking pathway of carbon cations, making C-C bonds more likely to undergo β-cleavage reactions and thereby improving the selectivity of gas products. In contrast, at a 15% Ag loading, the excess Ag may cover or shield some active sites, leading to acidity imbalance or pore blockage, reducing the number of effective catalytic sites and resulting in a decrease in cracking activity and conversion rate.

We carried out the activation energy analysis of the samples using the non-isothermal kinetic model proposed by Vyazovkin [39,40]. Equation (1) represents the integrated form of the kinetic model introduced by Vyazovkin. After obtaining the TGA data, the y-axis data were converted from residual mass percentage to conversion, with the specific conversion method shown in Equation (2), and the results are presented in Figure S6. Subsequently, the activation energy was calculated at 90% conversion. As shown in Equation (1), by plotting ln(β/$T_{a}^{2}$) versus 1/T_a_, the slope can be used to determine the activation energy [41], and the results are listed in Table S1. After the addition of ZSM-5, the activation energy decreased from 198.86 kJ·mol^−1^ to 171.99 kJ·mol^−1^, while further loading with 5% Ag led to a slight increase in activation energy. This trend is consistent with the results of catalytic cracking conversion and acidity analysis.

(1) $$Conversion=\frac{100-m_{1}}{m-m_{1}}\times 100$$

(2) $$\ln\left(\frac{\beta }{T_{a}^{2}}\right)=\ln\left[\frac{R\cdot k_{0}}{E_{a}\cdot g\left(a\right)}\right]-\frac{E_{a}}{R}\cdot \frac{1}{T_{a}}$$

where a is the conversion of the polymer degradation reaction: m is m represents the y-axis value in the TGA curve, m_1_ refers to the final residual mass, T_a_ is the temperature for reach to conversion a; b is the heating rate; R is universal gas constant; k_0_ is Arrhenius pre-exponential factor, E_a_ is the activation energy for a certain conversion a and g(a) is the integral of the kinetic model of reaction rate in function of the conversion. In most of the cases the function g(a) it does not present a defined form and after the model fitting, g(a) become implicit in the linear coefficient obtained.

Further analysis of the component distribution in the gaseous products from the catalytic cracking of XLPE using Ag-modified ZSM-5 nanosheets is shown in Figure 12b. It can be clearly observed that the introduction of Ag affects the selectivity towards light olefin products. In the catalyst without Ag loading, the total proportion of light olefins is 43.9%. As the Ag loading increases to 5%, the light olefin yield rises to 56.1%, reaching its peak, indicating that an appropriate amount of Ag helps improve the catalyst’s control over light olefins. Ag loading enhances the adsorption and dehydrogenation reactions of alkanes in the catalytic cracking process, promoting C-H bond cleavage, and thus improving the selectivity of gas products. However, when the Ag loading increases further to 15%, this proportion decreases to 50.4%, showing a trend of first increasing and then decreasing. This phenomenon may be due to the Ag loading modulating the surface acidity distribution of ZSM-5 and the synergistic effect at the metal–support interface. An appropriate amount of Ag loading helps adjust the strength and distribution of acidic sites on ZSM-5, making it more favorable for C-C bond β-cleavage and promoting the formation of light olefins. Additionally, Ag stabilizes the carbon cationic state in the reaction intermediates through charge redistribution at the metal–support interface, lowering the energy barrier for its dehydrogenation process, thus improving olefin selectivity. However, when the Ag loading exceeds a certain threshold (>5%), agglomeration between metals may occur, leading to partial coverage of acidic sites, while the pore structure may be hindered, limiting the diffusion and conversion efficiency of reactants and intermediates. Moreover, the excessive introduction of Ag may weaken the synergistic effect between the metal and the support, causing the catalytic reaction to gradually favor the thermodynamically more stable alkane product direction, resulting in a decrease in the light olefin yield.

Figure 12c shows the BTX distribution in the liquid products obtained from the catalytic cracking of XLPE using ZSM-5 nanosheets with different Ag loadings, as analyzed by GC. The BTX content in the liquid products from the catalytic cracking of XLPE using ZSM-5 nanosheets with different Ag loadings exhibits a trend of first decreasing and then increasing. Specifically, the BTX content in the Ag-free ZSM-5 nanosheets is 59.6%. With the increase in Ag loading, the BTX content in the 1% Ag-loaded sample decreases to 52.5%, but as the Ag loading continues to increase, the BTX content gradually increases and eventually stabilizes at about 60% in the 10% and 15% Ag-loaded samples. This phenomenon indicates that increasing Ag loading promotes the generation of BTX to some extent. Ag loading affects the catalytic cracking reaction path by modulating the distribution and strength of acidic sites in ZSM-5 nanosheets. As the Ag loading increases, the number of strong acid sites increases, thus promoting the formation of aromatics, especially BTX. At 1% Ag loading, due to the relative decrease in strong acid sites, the BTX proportion decreases. However, as the loading increases to 5% and higher, the increase in strong acid sites plays a positive role in the aromatization process of the cracking reaction, and therefore, the BTX content in the liquid products gradually rises to a higher level. Additionally, BET test data indicate that the pore structure of ZSM-5 nanosheets also plays an important role in the generation of aromatic compounds in the cracking products. Ag loading, by altering the pore size distribution of ZSM-5 nanosheets, further optimizes the product distribution of catalytic cracking.

We selected the solid powder obtained after the reaction of the catalyst with the best overall performance, 5%Ag-ZSM-5, and removed the residual unreacted XLPE by sieving with a 100-mesh sieve for TGA analysis of coke deposition. The heating rate was 10 °C/min under an air atmosphere. As shown in Figure S7, the weight loss observed between 30 and 120 °C was due to adsorbed water/volatiles on the sample surface. The weight loss in the range of 120–350 °C can be attributed to residual low-temperature oxidizable carbon, easily oxidizable alkyl groups/oligomers, and oxygen-containing carbon species in the catalyst. Subsequent weight loss corresponds to carbon black and refractory carbon. The results show that even when heated to 600 °C, the sample retained 96.05% of its original mass, indicating that no significant coke deposition occurred during the reaction.

Three repeated experiments were conducted to ensure the reproducibility of the results. As shown in Figure S8, there is no significant variation in either the conversion rate or the gas and liquid yields, with relatively small experimental errors, indicating good reproducibility. Five catalytic cracking cycles was using to the 5%Ag-ZSM-5 sample, which exhibited the best overall performance. The experimental procedure was as follows: after each reaction, the reactor and the solid residues were calcined in air at 500 °C for 1 h to obtain a solid powder. The calcined solid powder was then subjected to catalytic cracking again, and this cycle was repeated five times. The results are shown in Figure S9. After five cycles, the conversion, gas yield, and liquid yield remained nearly unchanged, at 92.16%, 80.65% and 11.51%, respectively. These cycling results demonstrate that the prepared 5%Ag-ZSM-5 possesses excellent regeneration capability and a long operational lifetime.

## 3. Conclusions

The ion-exchange method was used to load different metals onto ZSM-5 nanosheets, and the effects of metal loading on pore structure, acidity characteristics, and catalytic cracking performance were analyzed. The structure and acidity of the prepared samples were systematically analyzed using characterization techniques such as XRD, SEM, BET, and NH_3_-TPD, and their catalytic performance was evaluated in combination with Gas Chromatography (GC) detection. Based on the experimental results, the influence of different metal Ag loadings on the catalytic cracking performance of ZSM-5 nanosheets was further discussed. The main conclusions are as follows:

(1) The structural characterization results indicate that after loading different metals, the ZSM-5 nanosheets still retain the typical MFI-type molecular sieve structure, and the introduction of metals did not disrupt the original crystal structure. The metal ion loading altered the specific surface area and pore size distribution of the ZSM-5 nanosheets. In particular, the loading of Ce and Mo resulted in a decrease in both the specific surface area and mesopore volume. The impact of Ag loading on the specific surface area and mesopore volume was minimal. NH_3_-TPD tests indicated that metal ion loading enhanced the acidity of ZSM-5 nanosheets through metal–support interactions.

(2) Catalytic cracking experiments with ZSM-5 nanosheets loaded with different metal ions showed that metal ion loading enhanced the acidity of ZSM-5 nanosheets through metal–support interactions. Ag-loaded ZSM-5 nanosheets exhibited comparatively good catalytic activity and product distribution control capabilities, maintaining a conversion rate of 94.1%. The proportion of light olefins in the gas phase increased to 52.5%, while the proportion of BTX in the liquid products was 59.4%.

(3) Structural characterization results for ZSM-5 nanosheets with different Ag loadings (1%, 5%, 10%, 15%) showed that the Ag loading had no significant effect on the crystal structure of the ZSM-5 nanosheets, and Ag was evenly dispersed onto the framework and surface of the ZSM-5 nanosheets. The mesopore volume and average pore diameter of 5% Ag-ZSM-5 nanosheets reached their maximum values of 0.450 cm^3^/g and 5.52 nm, respectively, with the specific surface area remaining at 392.920 m^2^/g. As the Ag loading increased, the acidity enhanced, and the metal–strong acid interaction became stronger.

(4) Catalytic cracking experimental results for ZSM-5 nanosheets with different Ag loadings showed that 5% Ag-ZSM-5 nanosheets had comparatively good catalytic activity and product distribution control, maintaining a conversion rate of 92.3%. The proportion of light olefins in the gas phase reached 56.1%, and the proportion of BTX in the liquid products was 58.2%. This result indicates that an appropriate amount of Ag loading can optimize the distribution of acidic sites in ZSM-5 nanosheets, thereby promoting the formation of light olefins and maintaining the proportion of BTX products. The introduction of Ag improved the molecular cracking and aromatization processes in the catalytic cracking reaction by adjusting the acidity characteristics, thereby increasing the yield of target products.

## Figures and Tables

**Table 1:** Textural properties of metal-loaded ZSM-5 nanosheets.

Sample	S_BET_ (m^2^/g)	S_ext_^a^ (m^2^/g)	V_micro_^a^ (cm^3^/g)	V_mero_^b^ (cm^3^/g)	V_total_^c^ (cm^3^/g)	D_aver_^d^ (nm)
ZSM-5	419.440	62.395	0.154	0.477	0.580	0.822
10%Ni-ZSM-5	431.431	71.319	0.154	0.492	0.600	0.857
10%Ce-ZSM-5	392.462	63.624	0.144	0.333	0.427	0.798
10%Mo-ZSM-5	390.593	58.251	0.152	0.379	0.469	0.803
10%Ag-ZSM-5	392.621	65.866	0.141	0.419	0.502	0.812

^a^ Calculated by t-plot; ^b^ by BJH; ^c^ at P/P_0_ = 0.99; ^d^ pore diameter by DFT.

**Table 2:** Acidity of metal-loaded ZSM-5 nanosheets.

	Acid Site Concentration (µmol/g)			
	Weak-Medium	Strong	Metal-Strong	Total
ZSM-5	152.35	122.83	/	275.18
10%Ni-ZSM-5	159.46	80.98	193.44	433.88
10%Ce-ZSM-5	169.10	127.24	90.17	386.51
10%Mo-ZSM-5	139.16	133.36	83.41	355.93
10%Ag-ZSM-5	123.56	94.56	79.54	297.66

**Table 3:** Textural properties of ZSM-5 nanosheets with different Ag loadings.

Sample	S_BET_ (m^2^/g)	S_ext_^a^ (m^2^/g)	V_micro_^a^ (cm^3^/g)	V_meso_^b^ (cm^3^/g)	V_total_^c^ (cm^3^/g)	D_aver_^d^ (nm)
ZSM-5	419.440	62.395	0.154	0.477	0.580	0.822
1%Ag-ZSM-5	398.798	64.675	0.147	0.369	0.465	0.785
5%Ag-ZSM-5	392.920	68.711	0.143	0.450	0.542	0.819
10%Ag-ZSM-5	392.621	65.866	0.141	0.419	0.502	0.812
15%Ag-ZSM-5	393.797	63.145	0.144	0.387	0.483	0.796

^a^ Calculated by t-plot; ^b^ by BJH; ^c^ at P/P_0_ = 0.99; ^d^ pore diameter by DFT.

**Table 4:** Acidity of ZSM-5 nanosheets with different Ag loadings.

Sample	Acid Site Concentration (µmol/g)			
	Weak-Medium	Strong	Metal-Strong	Total
ZSM-5	152.35	122.83	/	275.18
1%Ag-ZSM-5	121.54	118.68	57.40	297.62
5%Ag-ZSM-5	116.81	105.54	70.14	292.49
10%Ag-ZSM-5	123.56	94.56	79.54	297.66
15%Ag-ZSM-5	141.31	87.54	90.14	318.99

## Data Availability

The original contributions presented in this study are included in the article/Supplementary Material. Further inquiries can be directed to the corresponding author.

## Supplementary Materials

The following supporting information can be downloaded at: https://www.mdpi.com/article/10.3390/ma18204675/s1, Figure S1. Schematic diagram of the synthesis steps of ZSM-5 nanosheets. Figure S2. Schematic diagram of the catalytic cracking device. Figure S3. Chromatogram of typical gaseous products. Figure S4. Chromatogram of typical liquid products. Figure S5. (a) XRD spectra of ZSM-5 loaded with different ratios of Ag ions and their SEM images: (b) 0%, (c) 1%, (d) 5%, (e) 10%, (f) 15%. Figure S6. Conversion data obtained from the TG curves with β = 5, 10, 20 ℃/min^−1^ of XLPE, XLPE + ZSM-5 and XLPE + 5%Ag-ZSM-5. Figure S7. TGA profile of 5% Ag-ZSM-5 after reaction. Figure S8. The conversion rate, gas yield, and liquid yield of three repeated experiments of (a) ZSM-5 nanosheets with different metal loadings, (b) ZSM-5 nanosheets with different Ag loadings. Figure S9. Conversion, gas yield, and liquid yield of 5%Ag-ZSM-5 over five cycles. Table S1. The Activation energies of XLPE, XLPE + ZSM-5 and XLPE + 5%Ag-ZSM-5. Table S2. XPS atomic ratios of ZSM-5 loaded with different proportions of Ag. Table S3. Gas chromatography analysis conditions for gaseous products. Table S4. Gas chromatography analysis conditions for liquid products.
