# Supplementary material for: The Use of Metal/ZSM-5 Nanosheet for Efficient Catalytic Cracking of Cross-Linked Polyethylene for High-Voltage Cable Insulation
Source: Materials (Basel). 2025 Oct 11;18(20):4675. doi: 10.3390/ma18204675 (PMC12565742; doi:10.3390/ma18204675)
Supplement: Supplementary file 1 [file materials-18-04675-s001.zip › materials-3891589-supplementary.pdf]

Supporting information

Metal/ZSM-5 Nanosheet as Efficient Catalysts Cracking of Cross-Linked Polyethylene for High Voltage Cable Insulation

Zhenfei Fu [a], Yuqi Pan[a], Rui Wang [a], Shilong Suo [a], Zhen Wang [b], Xiangyang Peng [b], Pengfei Fang [a,*]

[a] *School of Physics and Technology, Key Laboratory of Nuclear Solid State Physics Hubei Province, Wuhan University, Wuhan 430072, China*

[b] *Guangdong Key Laboratory of Electric Power Equipment Reliability, Electric Power Research Institute of Guangdong Power Grid Co., Ltd., Guangzhou 510080, China*

[a, *] *Corresponding author: Pengfei Fang*

Tel: +86 27 6875 2003;

Fax: +86 27 6875 2003.

E-mail address: fangpf@whu.edu.cn (Pengfei Fang)

Experimental section

1. Synthesis of samples

All reagents were used without further purification.

Preparation of the Precursor: Mix 50 mmol of TEOS and 18 mmol of TPAOH, and stir the mixture at 35°C for 5 hours, then stir at 45°C for 1 hour. Afterward, transfer the mixture to a high-pressure reactor and react at 70°C for 48 hours. This is labeled as "seed."

Preparation of ZSM-5: A molar ratio of 1:0.14:0.0025:0.8 for TEOS:TPAOH: $Al_2(SO_4)_3{\cdot}18H_2O$： NH_4F, with 50 wt% seed mixture, was used to prepare ZSM-5. Mix 50 mmol of TEOS and 7 mmol of TPAOH with 50 wt% seed mixture at 35°C for 1 hour to form solution A. Then, dissolve 40 mmol of NH_4F and 1.25 mmol of $Al_2(SO_4)_3{\cdot}18H_2O$ in deionized water and add them to solution A. Transfer the mixed solution to a high-pressure reactor and react at 170°C for 12 hours. The product is washed to neutrality with deionized water and anhydrous ethanol, then dried at 60°C for 12 hours and heated at 550°C for 6 hours.

Preparation of Metal-ZSM-5: Metal ion-loaded molecular sieves (denoted as Metal-ZSM-5) were prepared by ion-exchange. The selected metals include Ni, Mo, Ce, and Ag, with target loadings of 10 wt% for Ni, Mo, and Ce, and 1 wt%, 5 wt%, 10 wt%, and 15 wt% for Ag, respectively labeled as 1%Ag-ZSM-5, 5%Ag-ZSM-5, 10%Ag-ZSM-5, and 15%Ag-ZSM-5. The preparation steps are as follows: dissolve the appropriate amount of metal salt in deionized water to prepare a metal salt solution of the desired concentration. Then, add a certain amount of ZSM-5 nanosheet powder to the solution, maintaining a solution-to-catalyst ratio of 10 mL/g, and stir at room temperature for 3 hours. After the exchange,

centrifuge with deionized water and wash several times to remove any residual salts. Finally, dry the product at 80°C for 12 hours to obtain Metal-ZSM-5 nanosheets.

For specific synthesis steps, see Figure S1.

2. Characterization

The SmartLab SE X-ray diffraction (XRD, Rigaku, Japan) was performed in the 2θ range of 5°-55° with a scanning rate of 6°/min, using a D/teXUltra 250 silicon strip detector, Cu Kα radiation, with a working voltage of 40 kV and a current of 40 mA. The morphology and composition of the samples were characterized using a scanning electron microscope (SEM, HITACHI, Japan) equipped with an EDS detector (SU5000) and a transmission electron microscope (TEM, JEOL, Japan, JEM-2100). The valence states of the elements in the samples were analyzed using an ESCALAB 250Xi X-ray photoelectron spectrometer (XPS, Thermo Fisher, USA). Thermogravimetric (TG, Mettler-Toledo, Switzerland) experiments were performed with samples weighing less than 5 mg, placed in 80 μL alumina crucibles, using 99.999% pure nitrogen gas (N_2) as the inert atmosphere. The temperature was increased from 30°C to 800°C at a rate of 10°C/min, with the instrument model being TGA2. The specific surface area and pore size distribution were measured by the BET method (Beijing China GWGB, China), with the sample pretreated at 200°C for 6 hours. The instrument model was JW-BK122W. Ammonia temperature-programmed desorption (NH_3-TPD, Beijing China GWGB, China) was carried out by weighing approximately 0.1 g of the sample. Prior to testing, the sample was treated under a helium atmosphere at 400°C for 60 minutes. After the temperature was reduced to 100°C, 10% NH_3/He was introduced and maintained for 30 minutes to ensure full adsorption by the

sample. The flow was switched to helium (He) and purged for 60 minutes to remove excess He from the sample surface, after which the temperature was lowered to 50°C. Finally, the sample was heated to 800°C at a rate of 10°C/min, and the ammonia temperature-programmed desorption curve was obtained. The instrument model was AMI-300Lite.

3. Evaluation of Catalyst Catalytic Performance

In this study, XLPE catalytic cracking experiments were conducted using a laboratory-built cracking reaction device. During the experiment, 1500 mg of XLPE was thoroughly mixed with 300 mg of ZSM-5 nanosheet catalyst, and placed in the constant temperature zone of the reactor using a stainless steel basket (inner diameter 21 mm × length 50 mm). Before the reaction, the air inside the reactor was evacuated using a vacuum pump. The experiment was then conducted under a nitrogen flow rate of 35 mL/min, with the reactor being heated to 380°C at a rate of 10°C/min and maintained for 60 minutes. Liquid products were collected in a trap located below the reactor, while non-condensed gases were collected using an 8 L gas bag. The schematic diagram of the reaction device is shown in Figure S2.

After characterizing the catalyst, the product composition distribution was analyzed using gas chromatography (GC, Shangdon China SHISCO, China), with the instrument model being HF-901A. Non-condensed gaseous products were collected in an 8 L gas bag and analyzed by gas chromatography. The content of different components in the gaseous products was calibrated using the area normalization method with C_2H_4, C_3H_6, C_4H_8, and other components. The gas chromatography analysis conditions for the gaseous products are shown in Table 1, and the chromatograms of typical gaseous products analyzed under

these conditions are shown in Figure S3. The eluted compounds are as follows: methane (3.268 min), ethane (3.498 min), ethylene (3.788 min), propane (4.290 min), propylene (6.055 min), isobutane (6.557 min), n-butane (7.169 min), n-butene (11.042 min), trans-2-butene (11.262 min), isobutene (12.042 min), iso-pentane (12.674 min), and cis-2-butene (13.449 min).

Liquid products were collected after condensation. A ZY-5 capillary column was used to analyze the composition and content of the liquid products. The gas chromatography analysis conditions for the liquid products are shown in Table 2, and the chromatograms of typical liquid products analyzed under these conditions are shown in Figure S4. The typical eluted compounds are: benzene (13.992 min), toluene (17.797 min), and xylene (22.176 min).

Supplementary Results

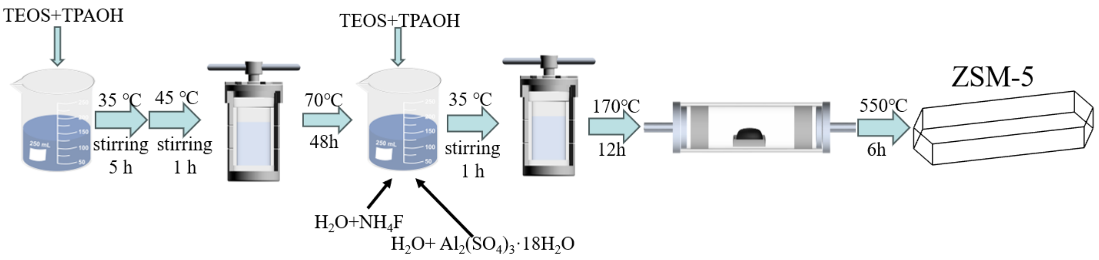

Figure S1. Schematic diagram of the synthesis steps of ZSM-5 nanosheets

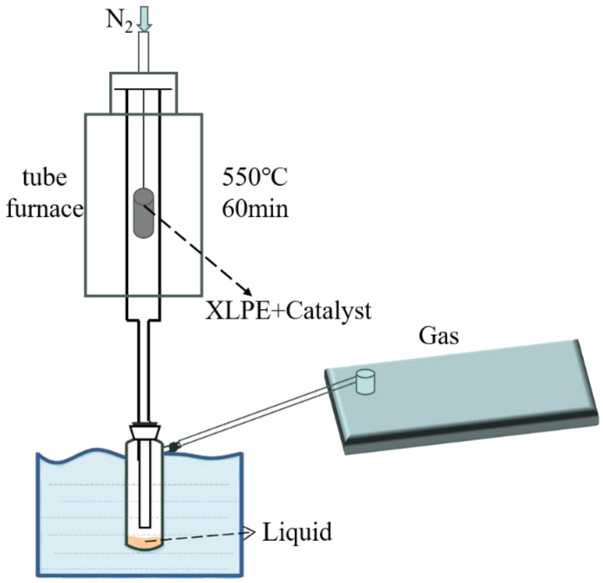

Figure S2. Schematic diagram of the catalytic cracking device

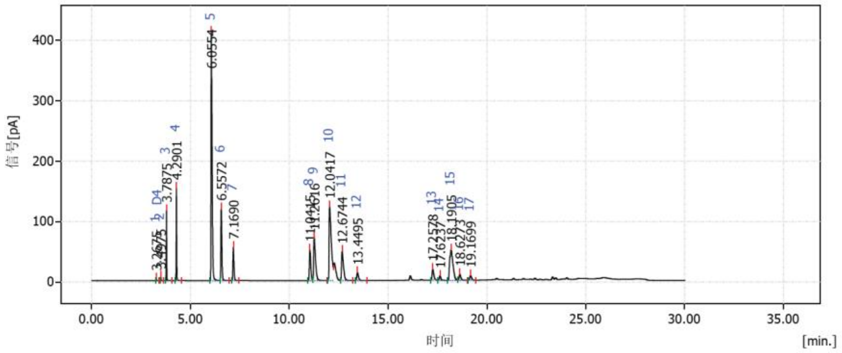

Figure S3. Chromatogram of typical gaseous products

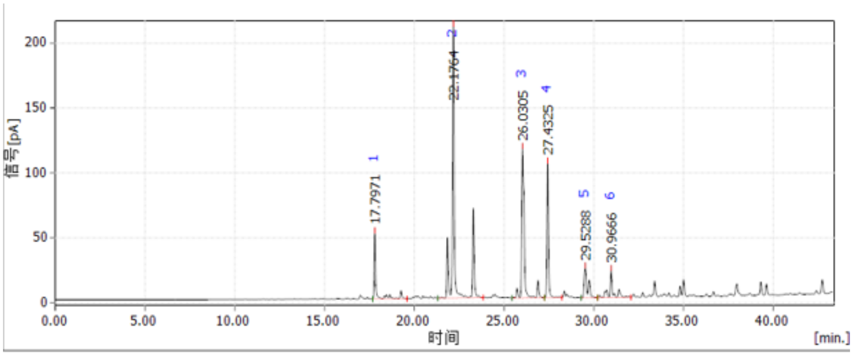

Figure S4. Chromatogram of typical liquid products

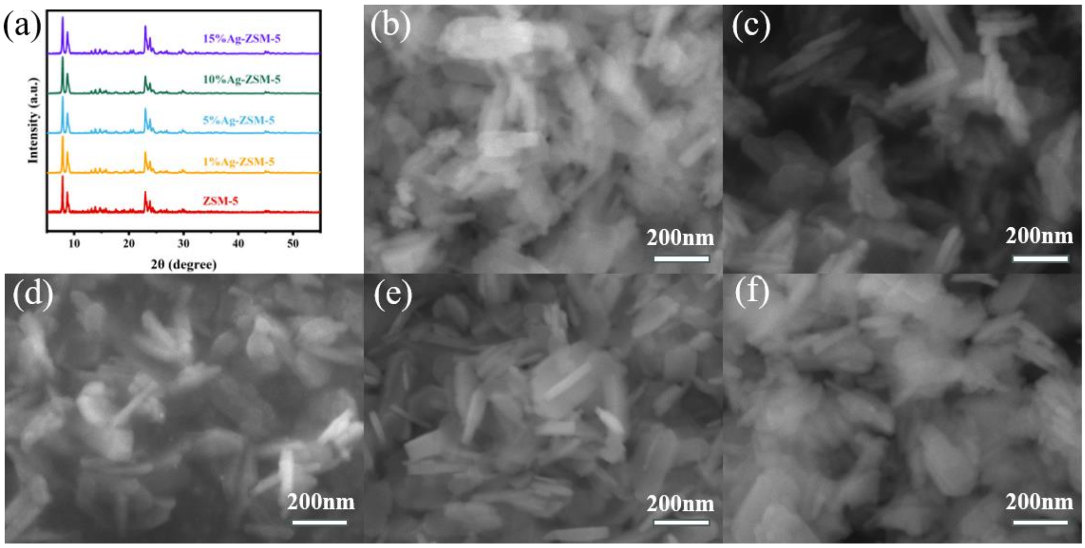

Figure S5. (a) XRD spectra of ZSM-5 loaded with different ratios of Ag ions and their SEM images: (b) 0%, (c) 1%, (d) 5%, (e) 10%, (f) 15%.

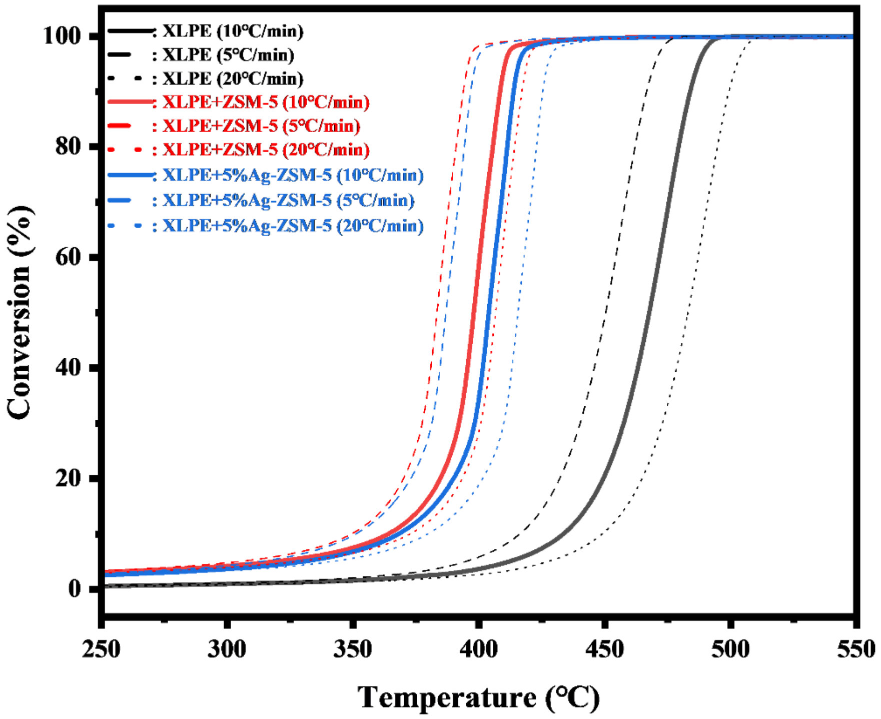

Fig. S6. Conversion data obtained from the TG curves with β=5, 10, 20 °C/min^{-1} of XLPE, XLPE+ZSM-5 and XLPE+5%Ag-ZSM-5.

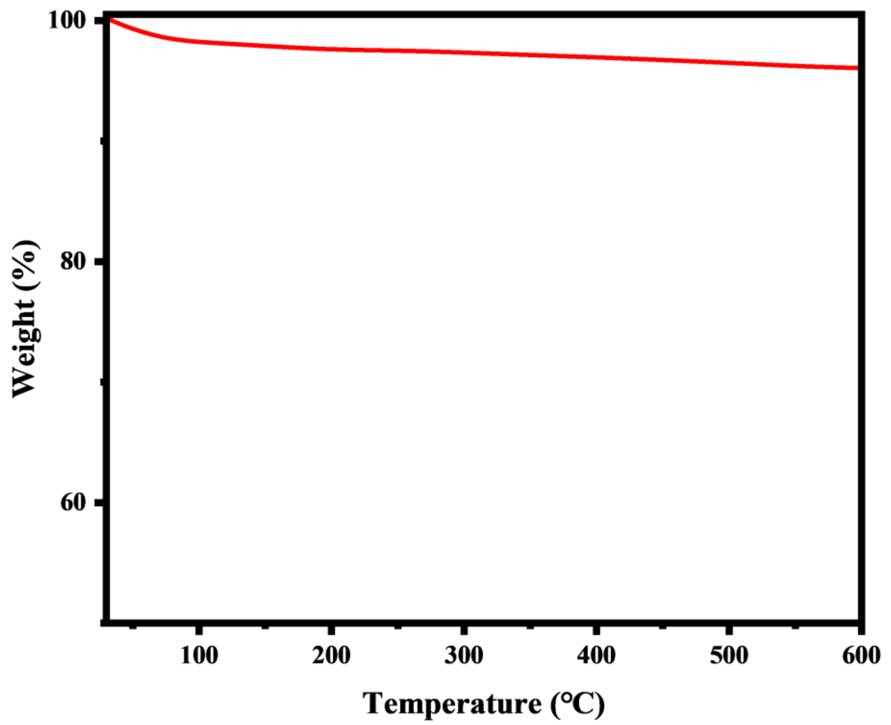

Figure S7. TGA profile of 5% Ag-ZSM-5 after reaction

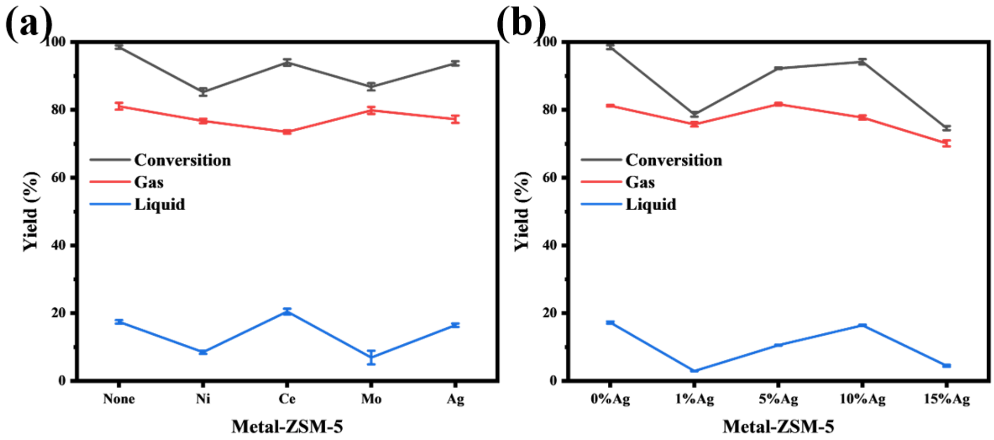

Fig. S8. The conversion rate, gas yield, and liquid yield of three repeated experiments of (a) ZSM-5 nanosheets with different metal loadings, (b) ZSM-5 nanosheets with different Ag loadings.

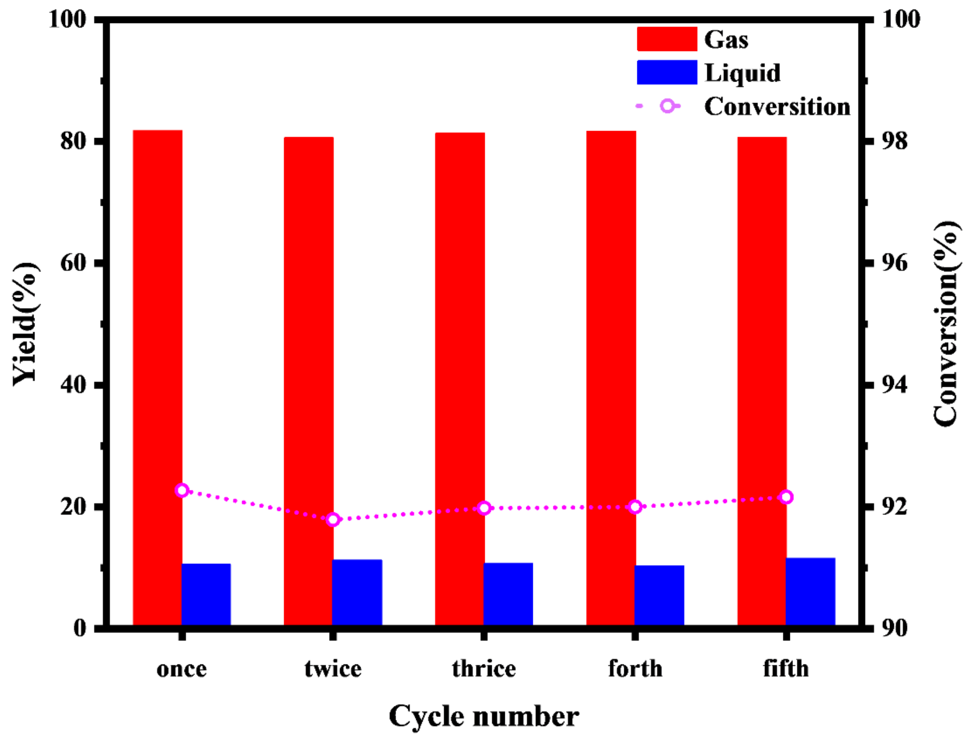

Fig. S9. Conversion, gas yield, and liquid yield of 5%Ag-ZSM-5 over five cycles.

Table S1. The Activation energies of XLPE, XLPE+ZSM-5 and XLPE+5%Ag-ZSM-5

Sample	XLPE	XLPE+ZSM-5	XLPE+5%Ag-ZSM-5
Activation energies ($kJ\cdot mol^{-1}$)	198.86	171.99	178.76

Sample	O (%)	Si (%)	Al (%)	Ag (%)
1%Ag-ZSM-5	65.66	32.69	1.53	0.12
5%Ag-ZSM-5	66.95	31.26	1.32	0.47
10%Ag-ZSM-5	66.21	32.17	1.00	0.62

15%Ag-ZSM-5	64.40	33.96	0.79	0.85

Table S2. XPS atomic ratios of ZSM-5 loaded with different proportions of Ag

Table S3. Gas chromatography analysis conditions for gaseous products

Name	GC
Detector	FID：180 °C
Chromatographic column	Al_2O_3/KCl，50 m×0.53 mm×20.0 μm
Temperature programming	Maintain at 50°C for 3 minutes, then heat at 5°C/min to 150°C and maintain for 5 minutes
Carrier gas	High-purity nitrogen gas

Table S4. Gas chromatography analysis conditions for liquid products

Name	GC
Detector	FID：280 °C
Chromatographic column	ZY-5，50 m×0.25 mm×0.5 μm
Temperature programming	Maintain at 50°C for 5 minutes, then heat at 5°C/min to 250°C and maintain for 10 minutes
Carrier gas	High-purity nitrogen gas
